# Serological correlates of disseminated *Borrelia burgdorferi* infection in mouse models of tick challenge

**DOI:** 10.1128/spectrum.01446-26

**Published:** 2026-08-28

**Authors:** Grace Freeman-Gallant, Muskan Shrestha, Stephanie You, Cecilia A. Silva-Valenzuela, Andrea Carfi, Meredith B. Finn, Christina Dold, Linden T. Hu, Nicholas Mantis

**Affiliations:** 1 Division of Infectious Diseases, New York State Department of Health 1094, Albany, New York, USA; 2 Department of Molecular Biology and Microbiology, Tufts University School of Medicine 12261, Boston, Massachusetts, USA; 3 Infectious Disease Research, Moderna, Inc., Cambridge, Massachusetts, USA; Creighton University, Omaha, Nebraska, USA; Tulane National Primate Research Center, Covington, Louisiana, USA

**Keywords:** antibody, complement, vaccine, transmission, protection

## Abstract

The mouse model of tick-mediated *Borrelia burgdorferi* sensu lato (*Bb*sl) challenge is integral to the preclinical evaluation of Lyme disease vaccines. The standard measure of vaccine efficacy is the recovery of viable spirochetes from different mouse tissues, monitored weekly for a month post challenge. Serological tests (ELISA and Western blots) with *B. burgdorferi* lysates, recombinant proteins, or peptides are often used as alternative or confirmatory readouts. While there are several reports suggesting that culture positivity and serology are strongly correlated, a systematic study addressing this issue is lacking. In this report, we took advantage of available data sets from large cohorts of naive and OspA actively and passively immunized mice that had been challenged with *Ixodes scapularis* nymphs carrying *Bb*sl strain B31, euthanized 3 weeks later, then assessed for culture positivity across different tissue types (ear, skin, ankle, bladder, and heart) and seropositivity using an OspC and DbpB 2-plex microsphere immunoassay (MIA) and a vmp-like sequence C6 monoplex MIA. Our results demonstrate a 96.5%–97% agreement between culture positivity and seroconversion using the OspC/DbpB and C6 MIAs, suggesting that serology provides a near-perfect readout of disseminated spirochetal infection. While some instances of culture and serological discordance could be attributed to technical errors, others likely reflect aspects of infectivity/immunological dynamics, including the possibility of abortive (non-sterilizing) *B. burgdorferi* infections. We conclude that serological testing provides a highly accurate and practical measure of disseminated *B. burgdorferi* infection in mouse models, crucial for the efficient preclinical assessment of Lyme disease vaccines.

**IMPORTANCE:**

The laboratory mouse (*Mus musculus*) plays an indispensable role in the testing of candidate vaccines for Lyme disease, an emerging tick-borne infection caused by the spirochete, *Borrelia burgdorferi*. While laboratory mice do not develop Lyme disease per se, *B. burgdorferi* proliferates in skin tissues following a tick bite, then disseminates to other organs over the course of days to weeks. In this report, we examined the concordance between two widely used methodologies to assess disseminated *B. burgdorferi* infection and offer recommendations to standardize testing across laboratories.

## INTRODUCTION

Lyme borreliosis is an emerging tick-borne disease caused by the spirochete, *Borrelia burgdorferi* sensu lato (*Bb*sl). In the United States alone, there are an estimated 450,000 cases of Lyme disease per year (1), and efforts to develop prophylactic vaccines and monoclonal antibodies are ongoing, particularly directed against outer surface protein A (OspA) (2, 3). Laboratory mice (*Mus musculus*) are widely used to model various aspects of human *B. burgdorferi* infection, including tick transmission, skin replication, and tissue dissemination (4, 5). Indeed, both tick-mediated and needle-inoculated *B. burgdorferi* challenge studies are an integral part of the preclinical testing of Lyme disease therapeutics and vaccines (6–9). While certain inbred mouse strains develop joint pathology that is measurable in tissue sections, mice do not develop Lyme disease or generally display any overt signs of infection (10). That raises the question of how to best assess infectivity when evaluating the efficacy of Lyme disease vaccines in murine models.

The gold standard for assessing *B. burgdorferi* dissemination in the laboratory mouse (*Mus musculus*) is the recovery in culture of viable spirochetes from one or more tissues at necropsy. False negatives are a potential concern because of the fastidious nature of *B. burgdorferi* and inherent challenges associated with tissue collections and visual (microscopic) inspections of cultures for viable spirochetes over many weeks. For that reason, serological assays to detect *B. burgdorferi*-specific antibodies are frequently run in parallel, with a positive outcome by either method (culture or serology) considered sufficient proof of infection. Indeed, spirochete infection is associated with a robust serological response to an array of *B. burgdorferi* outer surface proteins, including outer surface protein C (OspC), decorin binding proteins A (DbpA) and B (DbpB), vmp-like sequence (VlsE), and VlsE-derived peptides like C6 (11–13). In fact, current laboratory-based Lyme disease diagnostic assays for humans are based on multi-antigen and often multi-tiered serological assays (14). Some investigators have argued that in laboratory mice, IgG serology is equal to, if not more sensitive and reliable than, culturing spirochetes from one or more organs (15, 16). However, a definitive report documenting the relationship between culture positivity and serology in experimentally challenged animals, particularly in the context of passive and active immunization with next-generation OspA vaccines, is lacking.

## RESULTS AND DISCUSSION

To address this gap, we analyzed the concordance between culture positivity and seroconversion in cohorts of naive (*n* = 49) and OspA-vaccinated (*n* = 205) female C3H/HeN mice (Charles River, Wilmington, MA) that had been challenged with *Ixodes scapularis* nymphs infected with *B. burgdorferi* sensu stricto strain B31 (Table 1) (17, 18). Culture positivity results from three different tissue categories (ear and skin [ES], ankle and bladder [AB], and heart [H]). Tissue samples for *B. burgdorferi* culture were obtained from skin (2 mm punch biopsies), the entire bladder, ankles (bone segments ~3 mm proximal and distal to the joint after skin removal), and the cardiac apex (3 mm segment). Tissues were immersed in BSK II medium containing rifampicin (50 μg/mL), fosfomycin (20 μg/mL), and amphotericin B (2.5 μg/mL) and placed in an incubator with 5% CO_2_ environment. Cultures were monitored twice weekly for 4 weeks by dark-field microscopy for evidence of growth of spirochetes. Tick challenge studies were conducted in the same research laboratory under the same conditions.

**TABLE 1:** Distribution of culture and serology positivity in mouse cohorts following *B. burgdorferi* tick-mediated challenge

Cohort	Culture/serology status				Total (*n*)
	+^*a*^/+^*b*^	−/−	+/−	−/+	
Naive	48 (98%)	1 (2%)	0	0	49
Vaccinated	17 (8%)	179 (87%)	6 (3%)	3 (1.5%)	205

^*a*^ + indicates at least one tissue category was positive.

^*b*^ + indicates DbpB/OspC index >1.

The results from the *B. burgdorferi* tissue cultures were compared to results obtained by an IgG serological 2-plex microsphere immunoassay (MIA) consisting of recombinant OspC type A (OspC_A_) and DbpB from strain B31 (DbpB_B31_). Within the OspC_A_/DbpB_B31_ 2-plex, a combined index value of greater than one was classified as seropositive. The combined index value is defined as the mean OspC_A_ and DbpB_B31_ indexes, which were derived by dividing the antigen-specific median fluorescent intensity (MFI) values (enumerator) by 6 times the buffer-only MFI values (denominator).

**Fig 1:**
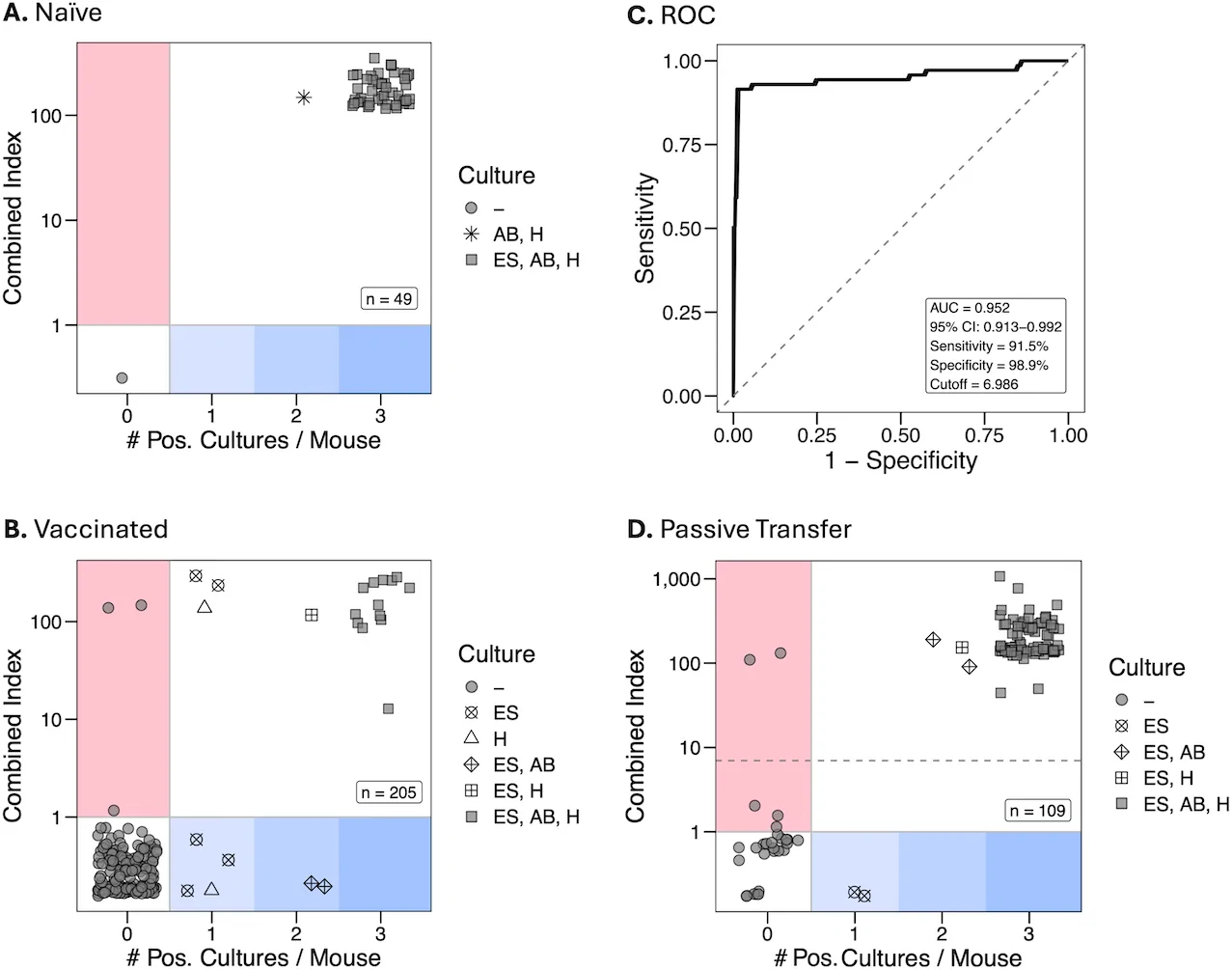
Comparison of serological and culture results for naive and actively and passively OspA immunized *B. burgdorferi* (B31) challenged mice. Serum from naive (**A**, *n* = 49) and OspA-vaccinated (**B**, *n* = 205) mice was subjected to a microsphere immunoassay using a panel of *B. burgdorferi* antigens to determine infection status. MFIs for OspC_A_ and DbpB_B31_ were indexed using a seropositivity threshold defined as 6× mean buffer-only MFI and averaged to calculate the combined index measure (*y*-axis). A combined index value greater than 1 indicates seroconversion for the given antigen. The number of cultures positive per mouse is shown on the *x*-axis, with a sample having one or more positive cultures indicating infection. Each point represents a single sample, and the shape indicates the identity of the positive culture(s) (ear and skin [ES] coculture, ankle and bladder [AB] coculture, heart [H], or any combination of the three). Points falling in the pink-shaded region show samples that were positive by the 2-plex serology measure but negative by culture (c^−^/s^+^), and those falling in the blue-shaded region show samples that were positive by culture and negative by serology (c^+^/s^−^). Samples falling in the unshaded region of the plot are either “double positive” or “double negative,” showing that the two methods of infection detection aligned. (**C**) Receiver operating characteristic (ROC) curve showing the ability of serology results to model culture outcomes (AUC = 0.95 [95% CI 0.91–0.99]). Youden’s *J* statistic was calculated as an optimal seropositivity threshold for use in future serology assays. (**D**) Serum from passively immunized (*n* = 109) mice was subjected to the OspC_A_/DbpB_B31_ 2-plex and indexed as previously stated. The gray horizontal dashed line represents the ideal seropositivity threshold established by the ROC analysis using the naive and vaccinated cohort (*y* = 7).

In the unvaccinated cohort, there was 100% agreement between culture positivity and serology, with 48 “double-positive” mice (positive for both culture and serology) and a single “double-negative” mouse (Table 1). In the double positive group, 47 of the 48 mice were culture positive for all three tissue groups (ES, AB, and H), while a single mouse was positive for AB and H but not ES (Fig. 1A). Thus, there was essentially perfect concordance between serology and culture positivity. Moreover, the observation that ES tissue cultures captured infectivity in all but one mouse suggests that ES are the tissues of choice for assessing bacterial dissemination.

In the OspA-vaccinated cohort of mice (*n* = 205), there was a 95.6% agreement between culture and serology, with 179 (87%) classified as double negative (c^−^/s^−^) and 17 (8%) classified as double-positive (c^+^/s^+^) (Table 1). Nine mice (4.4%) had discordant outcomes: six were culture positive/serology negative (c^+^/s^−^, blue shading; Fig. 1B), and three were culture negative/serology positive (c^−^/s^+^, red shading; Fig. 1B). Within the total population of both naive and actively immunized mice (*n* = 254), culture and serology aligned 96.5% of the time (*κ* = 0.91, 95% CI 0.85–0.97).

Using the total naive and vaccinated sample set (*n* = 254), we performed receiver operating characteristic (ROC) analysis to establish the threshold at which the serological combined index most accurately classifies culture positivity. Culture positivity was plotted on the *y*-axis and serological outcome on the *x*-axis. ROC analysis resulted in an area under the curve (AUC) of 0.95 (95% CI 0.91–0.99), indicating that the OspC_A_/DbpB_B31_ 2-plex is nearly perfect at classifying culture positivity. Application of Youden’s *J* statistic post hoc test indicated that the optimal threshold for both sensitivity and specificity corresponds to a combined index of 7 (Fig. 1C). The inclusion of additional recombinant *B. burgdorferi* antigens (e.g., OspC types, DbpA, etc.) in the 2-plex MIA did not improve the overall performance of the assay even though others have observed increased sensitivity with as many as five target antigens (19).

To validate the use of the optimized serological threshold, we next applied the post hoc analysis to a third cohort of mice (*n* = 109) that had been passively administered OspA antisera prior to challenge with *I. scapularis* nymphs carrying *B. burgdorferi* B31 (Fig. 1D). In this data set, there was 96.3% agreement between culture and serology, with 79 (72%) classified as double positive and 26 (24%) classified as double negative. Three mice that were originally classified as seropositive using the arbitrary sixfold over background (blank) cutoff were re-classified as serology negative based on the ROC-derived threshold, thereby improving concordance with culture outcome (Fig. 1D, pink shading below the horizontal dashed line). In the end, there were just four mice (3.7%) with discordant outcomes (two c^+^/s^−^ and two c^−^/s^+^). These results demonstrate that the higher cutoff derived from ROC analysis minimizes type 1 errors and retains optimal assay sensitivity.

### Concordance between culture positivity and C6 serology

The VlsE-derived C6 peptide and C6 derivatives are commonly used as serological markers for Lyme disease in human diagnostic tests and occasionally as the sole indicator of *B. burgdorferi* infection in animal studies, including preclinical OspA vaccine reports (7, 8, 20, 21). C6 is an appealing serological reagent because it is derived from a highly conserved region within the so-called invariable region 6 of VlsE (11, 12, 22). However, it should be noted that C6 corresponds to an internal (buried) epitope that is presumably only accessible to B-cell receptor recognition when VlsE is partially unfolded or degraded after being shed from the bacterial surface, possibly at points after immune responses have been elicited against DbpB and OspC (11).

We therefore examined the performance of a VlsE C6 17-mer derived from strain B31 (MKKDDQIAAAIALRGMA) (12, 23) as a stand-alone serological measure of disseminated infection and compared its performance to the OspC_A_/DbpB_B31_ 2-plex MIA. A total of 283 samples from the existing cohorts of naive, OspA-vaccinated, and passively immunized mice were tested for C6 reactivity and indexed as previously described. C6 reactivity (as determined by MFI) was associated with considerably more background relative to the 2-plex, with a mean index value (±SD) of 2.1 ± 3.8 for the double-negative samples (*n* = 203) compared to 0.4 ± 0.2 for OspC_A_ and 0.3 ± 0.1 for DbpB_B31_. There was also much greater inter-assay variability, making it difficult to resolve a common threshold for seropositivity determination, an observation noted by others in the literature (24).

**Fig 2:**
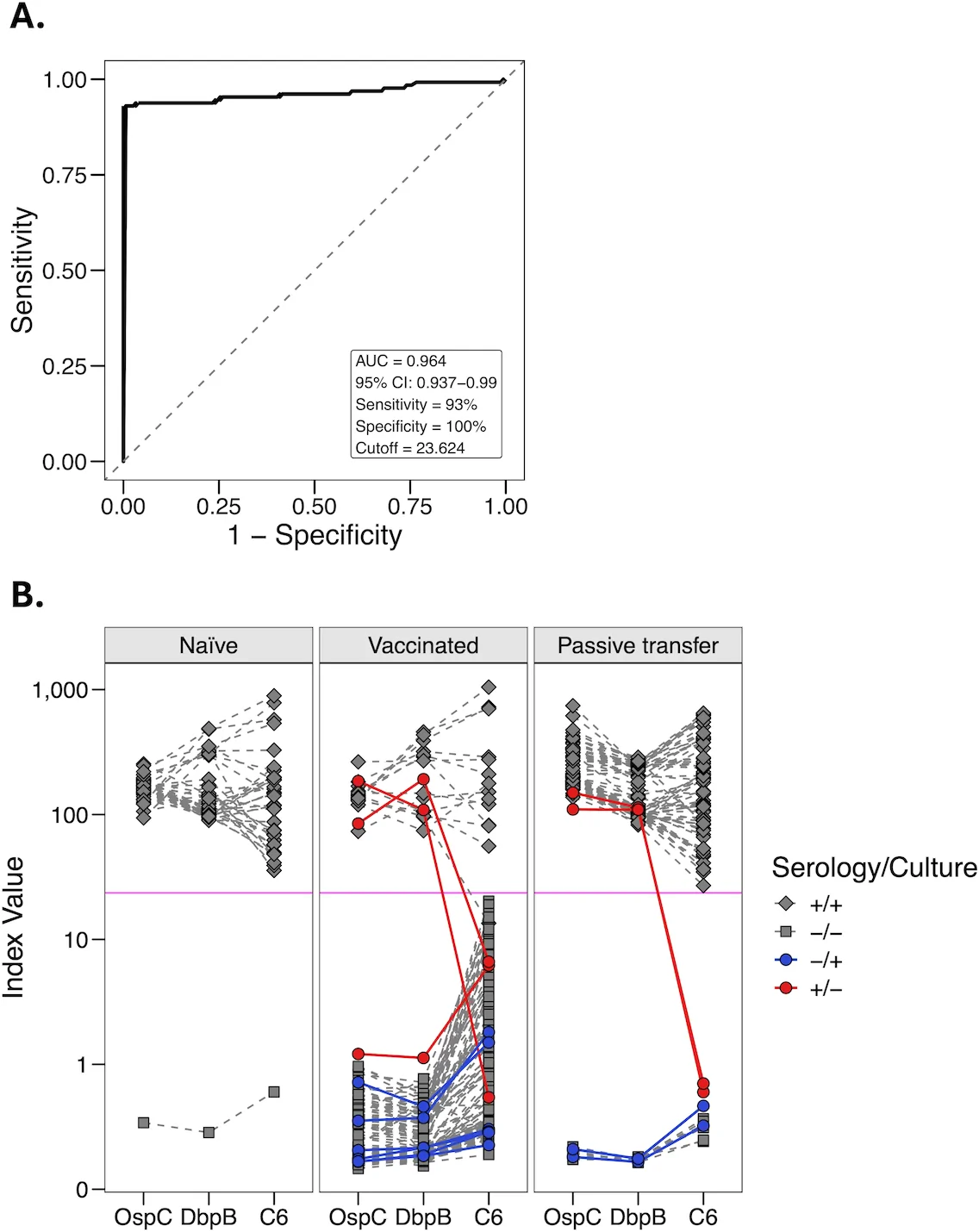
Using C6 monoplex and OspC_A_/DbpB_B31_ 2-plex to measure serology in comparison to culture. ROC analysis (**A**) was conducted to assess the ability of the C6 monoplex to model infection results determined by culture using serum from a cohort of naive and actively and passively OspA immunized mice (*n* = 283) (AUC = 0.96 [CI 0.94–0.99]). Youden’s *J* statistic was calculated as an optimal seropositivity threshold by which to interpret individual sample results in further analysis. (**B**) The two methods of serology (OspC_A_/DbpB_B31_ 2-plex and C6 monoplex) were compared. MFIs for OspC_A_, DbpB_B31_, and C6 were indexed as previously described and plotted with the antigen on the *x*-axis and index value on the *y*-axis. Each point is representative of an individual index measurement with lines connecting indices that belong to a single serum sample. The lines are colored based on the culture/serology profile previously determined by the OspC_A_/DbpB_B31_ 2-plex, with gray showing either “double-positive” or “double-negative” samples where culture and serology aligned, red indicating a c^−^/s^+^ result, and blue indicating a c^+^/s^−^ result. The pink horizontal line shows the seropositivity cutoff as determined using the ROC analysis above (*y* = 23.6). C6 indices falling above this threshold were considered positive by this assay.

Nonetheless, we conducted a post hoc ROC analysis using C6 indexes and corresponding culture outcomes. The C6 index value proved to be an excellent determinant of culture positivity (AUC = 0.96 [95% CI 0.94–0.99]) (Fig. 2A). An optimal seropositivity cutoff of 23.6 was determined using Youden’s *J* statistic and subsequently applied to the sample set. By this method, C6 serology aligned 96.8% of the time with culture positivity (*n* = 274). In all but six instances, the serology determination by C6 was identical to that of the OspC_A_/DbpB_B31_ 2-plex (98% concordance) (Fig. 2B). Interestingly, C6 indexing did not misclassify any samples as serology positive in mice that were culture negative. By comparison, the combined OspC_A_/DbpB_B31_ 2-plex index classified 3 of 254 (1.2%) of the samples as being serology positive when the animals were classified as culture negative. When considering the reverse scenario, the C6 (3.2%) and OspC_A_/DbpB_B31_ 2-plex (2.4%) indices had a similar number of samples that were classified as serology negative despite the animals being culture positive. These results indicate that, when empirically defined thresholds are applied, C6 and OspC_A_/DbpB_B31_ are essentially equivalent readouts of disseminated *B. burgdorferi* infection in the C3H mouse model.

### Nature of the discordance between serology and culture positivity

**Fig 3:**
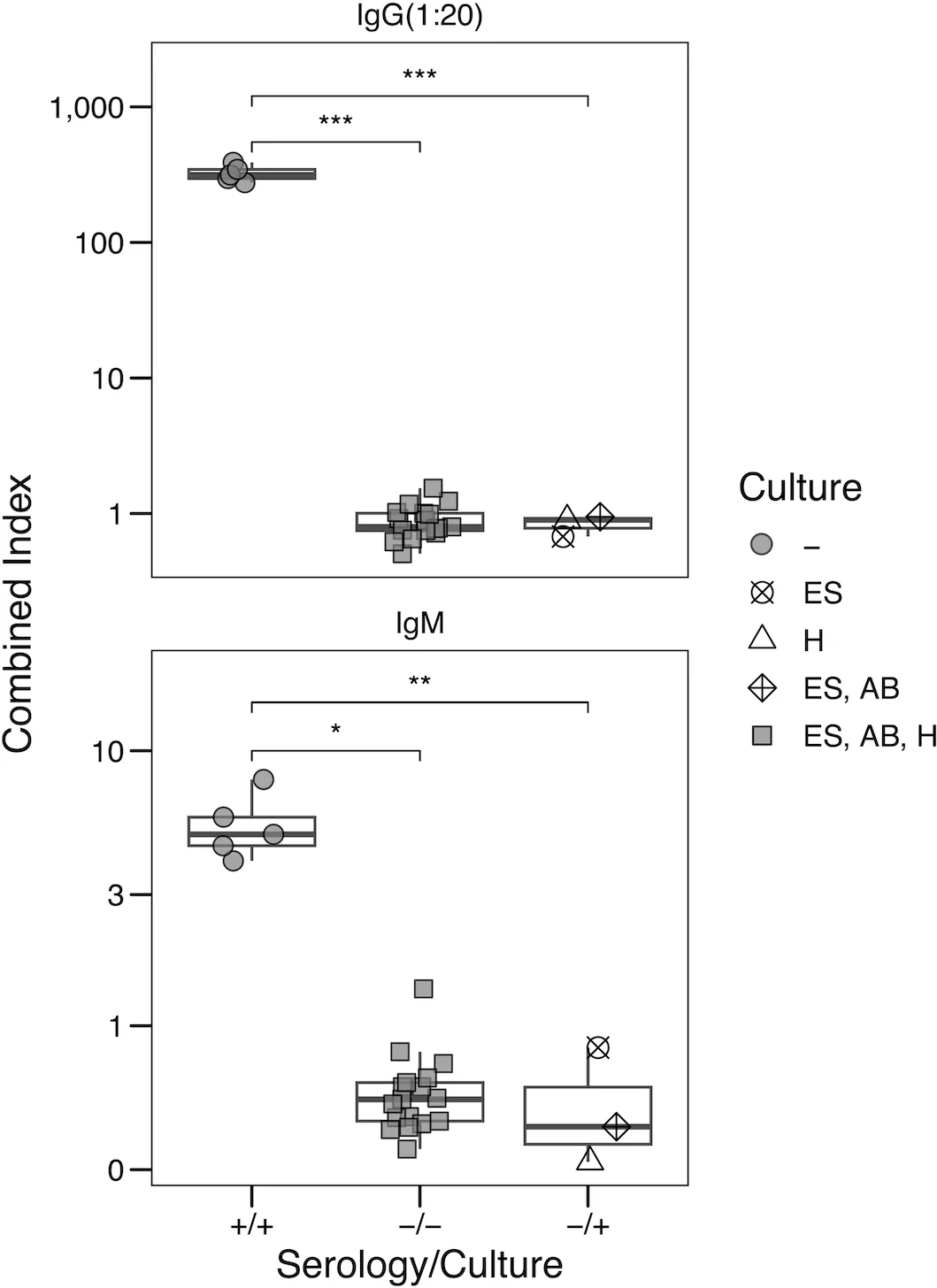
Further serological analysis of c^+^/s^−^ samples. A subset of c^+^/s^−^ samples (*n* = 3) was subjected to the OspC_A_/DbpB_B31_ 2-plex MIA at a lower dilution (1:20) and probed for IgG reactivity that was missed at the standard 1:100 dilution (top). Additionally, a 1:100 serum dilution was probed using an IgM secondary (bottom). MFIs for each antigen were indexed as previously seen and plotted on the *y*-axis. The serum samples in this study were divided based on their culture/serology profile as previously determined by the OspC_A_/DbpB_B31_ 2-plex and plotted on the *x*-axis. The shape of the point indicates which (if any) cultures/cocultures were positive. Statistical differences between groups were calculated using a *t*-test with Bonferroni *P* value adjustment (*** <0.001; ** <0.01; * <0.05).

Within the OspA-vaccinated group of mice analyzed by the OspC_A_/DbpB_B31_ 2-plex, there were six discordant samples (2.9%) classified as culture positive but serology negative (c^+^/s^−^) (Fig. 1B, highlighted in blue). All six of those samples were also negative by C6 MIA. The serology-negative (s^−^) classification was not due to a lack of assay sensitivity, as the serum samples were still negative when re-tested at lower dilutions (1:20) in the 2-plex MIA for IgG and when evaluated for *B. burgdorferi*-specific IgM at 1:100 (Fig. 3). It is therefore tempting to speculate that the discordance is biologically relevant in that spirochetal infection went undetected by the humoral immune system, possibly because bacterial replication was confined within peripheral tissues (e.g., skin). Consistent with this idea, culture positivity in three of the six mice was limited to ES. However, the other three serology-negative mice had disseminated infections, as evidenced by recoverable spirochetes in AB and H + ES, indicating that discordance may in fact be due to other factors.

There were three discordant samples (1.5% of total) in the OspA-vaccinated group of mice classified as serology positive by the 2-plex MIA but culture negative (Fig. 1B, highlighted in pink). Interestingly, the three discordant samples were also serologically negative for C6 (Fig. 2B). We postulate that this profile (i.e., 2-plex^+^, C6^−^, and culture^−^) is indicative of an abortive infection in which there may have been sufficient antigenic exposure to drive OspC and DbpB seroconversion, but clearance of the spirochetes before VlsE onset (25–27). As noted above, C6 antibodies target a cryptic epitope on VlsE that would arise only in response following VlsE shedding from the bacterial surface or bacterial lysis (11). Considering that the 2-plex^+^, C6^−^, and culture^−^ profile was observed only in OspA-vaccinated mice and not in naive mice, we would postulate that clearance may be driven by OspA antibody titers below the threshold for sterilizing immunity but at levels sufficient to attenuate challenge dose. Alternatively, the early onset of OspC and/or DbpB antibodies themselves may have been responsible for spirochete clearance through complement or Fc receptor-mediated functions. In either case, it is interesting to speculate that certain serological profiles may reflect degrees of bacterial infection.

### Conclusions

The importance of the mouse model of tick-mediated *Bb*sl challenge in the preclinical evaluation of Lyme disease vaccines has been recognized for decades (28). It is therefore surprising that a standard agreed-upon method to assess spirochete infectivity and dissemination in laboratory mice has not been universally adopted. In fact, a definitive study examining the exact relationship between two of the most common readouts—serological assays based on recombinant antigens or peptides and spirochete culture positivity from different tissues collected following tick-mediated challenge—is lacking. In this report, we took advantage of available data sets from large cohorts of naive and OspA actively and passively immunized mice that had been challenged with *I. scapularis* nymphs carrying *Bb*sl strain B31 to examine the relationship between culture status across different tissue types (ear, skin, ankle, bladder, and heart) and serological status based on two different MIAs, OspC/DbpB and C6. Within this data set, our results demonstrated a “near-perfect” agreement between culture and serological readouts in both naive and immunized (active and passive) groups of mice. Discordant outcomes were infrequent.

While it could be argued that one method alone is sufficient for use in preclinical vaccine testing, we would make the case that as a standard of practice, both methods should be performed and reported when making claims about preclinical vaccine efficacy. Our results indicate that collection of just the skin/ear tissues (not other organs or tissues) for culture assessment is the most sensitive readout. Moreover, a C6 MIA or possibly an ELISA would be a sufficient measure of seroconversion in both localized and disseminated infections as it most closely replicates culture readouts, with the stipulation that necessary thresholding has been conducted. In fact, based on the results presented in Fig. 1 and 2, using the C6 peptide and ES coculture would have resulted in only one (0.4%) missed infection. The inclusion of an OspC_A_/DbpB_B31_ 2-plex may capture protection in the absence of sterilizing immunity, a profile that is missed by C6 MIA and culture readouts. The inclusion of qPCR for the detection of spirochete DNA from ear or bladder alongside the other two metrics would add an additional layer of confirmation and possibly quantitation of bacterial burdens (29).

This study is subject to several limitations, most notably the use of a single challenge strain (B31) and regimen, in a single mouse species with a limited antigen repertoire. In vaccinated mice, bacterial burdens may be significantly reduced, making them even harder to recover in culture leading to a higher rate of false negatives. We also acknowledge that different in-house serological tests have varying thresholds that need to be established and that serological tests such as those described herein are most relevant for vaccines that aim to prevent infection entirely and are less pertinent in the case of vaccines that promote clearance of *B. burgdorferi* after early infection.

## MATERIALS AND METHODS

### Mouse immunizations and tick challenges

Serum samples used in this article were derived from animal studies conducted elsewhere (17). In those studies, female C3H/HeN mice (Charles River) were immunized intramuscularly in the hind leg quadriceps with experimental OspA vaccine formulations, then challenged with *Ixodes scapularis* nymphs infected with *B. burgdorferi* strain B31 (18). The mice were euthanized 2 weeks following the recovery of the last engorged tick, and the following tissues were collected: skin biopsy at the bite site, heart, ear, ankle, and bladder. A sample tissue of each of these organs was placed into Barbour-Stonner-Kelly media supplemented with rifampicin, amphotericin B, and phosphomycin and incubated at 37°C. The cultures were monitored twice weekly for viable spirochetes by darkfield microscopy for up to 4 weeks. Animal groups and culture positivity readouts are provided in Data set S1.

### MIAs

Recombinant OspC (type A, residues 38–201, AAC66329.1 GenBank) and DbpB (residues 21–187, UniProt ID P0CL68) were kindly provided by Dr. Michael Rudolph (New York Structural Biology Center). OspC and DbpB were coupled to Magplex-C microspheres (5 μg antigen/1 × 10^6^ microspheres) using an xMap Antibody Coupling Kit as recommended by the manufacturer (Luminex Corporation, Austin, TX). For Luminex assays, microspheres (diluted 1:50, 50 μL) and sera (diluted 1:100, 50 μL) were combined in black, clear-bottomed, non-binding, chimney 96-well plates (Greiner Bio-One, Monroe, NC) and incubated at room temperature for 1 h in a tabletop shaker. Plates were washed (1× PBS, 2% BSA, 0.02% Tween-20, 0.05% sodium azide, pH 7.4) using a magnetic separator, then probed with goat antimouse IgG, human-ads-PE (SouthernBiotech, Birmingham, AL) diluted 1:500. Plates were analyzed using a FlexMap 3D (Luminex Corporation). VlsE-derived C6 B31-17 peptide (MKKDDQIAAAIALRGMA) (12) with a GGGSK-biotin linker was synthesized and complexed to MagPlex-avidin microspheres as described (23).

### Statistical analysis

To establish reactivity cutoffs for OspC_A_ and DbpB_B31_, the average MFI of buffer-only wells was multiplied by six. MFIs for each mouse serum sample were divided by the antigen-specific reactivity cutoffs, yielding an index value. An index value >1 is indicative of above-background reactivity for a given antigen. Index values for samples used in this study are provided in Data set S1. Further analysis was completed using R 4.4.1 with R packages readx (30), tidyverse (31), ggplot2 (32), ggthemes (33), dplyr (34), rstatix (35), ggh4x (36), and ggpubr (37). Receiver operating characteristic analysis was completed using the pROC package (38).

## Supplementary Material

Reviewer comments

## SUPPLEMENTAL MATERIAL

Data set S1 spectrum.01446-26-s0001.xlsx Serology and culture results for all mice included in the study.

OPEN PEER REVIEW reviewer-comments.pdf An accounting of the reviewer comments and feedback.

ASM does not own the copyrights to Supplemental Material that may be linked to, or accessed through, an article. The authors have granted ASM a non-exclusive, world-wide license to publish the Supplemental Material files. Please contact the corresponding author directly for reuse.
