# Supplementary material for: Serological correlates of disseminated Borrelia burgdorferi infection in mouse models of tick challenge
Source: Microbiol Spectr. 2026 Aug 28;14(10):e01446-26. doi: 10.1128/spectrum.01446-26 (PMC13688919; doi:10.1128/spectrum.01446-26)
Supplement: Supplementary file 1 [file reviewer-comments.pdf]

ASM | Microbiology Spectrum

Serological correlates of disseminated *Borrelia burgdorferi* infection in mouse models of tick challenge

Grace Freeman-Gallant, Muskan Shrestha, Stephanie You, Cecilia Silva-Valenzuela, Andrea Carfi, Meredith Finn, Christina Dold, Linden Hu, and Nicholas Mantis

Corresponding Author(s): Nicholas Mantis, Wadsworth Center, New York State Department of Health

Review Timeline:

Submission Date:	May 28, 2026
Editorial Decision:	June 24, 2026
Revision Received:	August 3, 2026
Accepted:	August 4, 2026

Editor: Travis Bourret

Reviewer(s): Disclosure of reviewer identity is with reference to reviewer comments included in decision letter(s). The following individuals involved in review of your submission have agreed to reveal their identity: Monica E. Embers (Reviewer #2)

Transaction Report:

(Note: With the exception of the correction of typographical or spelling errors that could be a source of ambiguity, letters and reports are not edited. The original formatting of letters and referee reports may not be reflected in this compilation.)

DOI: https://doi.org/10.1128/spectrum.01446-26

Re: Spectrum01446-26 (**Serological correlates of disseminated *Borrelia burgdorferi* infection in mouse models of tick challenge**)

Dear Dr. Nicholas J. Mantis:

Thank you for the privilege of reviewing your work. Below you will find my comments, instructions from the Spectrum editorial office, and the reviewer comments.

The comments from reviewer 2 are minor in scope and only require textual modifications.

Please return the manuscript within 60 days; if you cannot complete the modification within this time period, please contact me. If you do not wish to modify the manuscript and prefer to submit it to another journal, notify me immediately so that the manuscript may be formally withdrawn from consideration by Spectrum.

Revision Guidelines
To submit your modified manuscript, log into the submission site at https://spectrum.msubmit.net/cgi-bin/main.plex. Go to Author Tasks and click the appropriate manuscript title to begin. The information you entered when you first submitted the paper will be displayed; update this as necessary. Note the following requirements:

• Upload point-by-point responses to the issues raised by the reviewers in a file named "Response to Reviewers," NOT in your cover letter.
• Upload a compare copy of the manuscript (without figures) as a "Marked-Up Manuscript" file.
• Upload a clean .DOC/.DOCX version of the revised manuscript and remove the previous version.
• Each figure must be uploaded as a separate, editable, high-resolution file (TIFF or EPS preferred), and any multipanel figures must be assembled into one file.
• Any supplemental material intended for posting by ASM should be uploaded with their legends separate from the main manuscript. You can combine all supplemental material into one file (preferred) or split it into a maximum of 10 files with all associated legends included.

For complete guidelines on revision requirements, see our Submission and Review Process webpage. Submission of a paper that does not conform to guidelines may delay acceptance of your manuscript.

Data availability: ASM policy requires that data be available to the public upon online posting of the article, so please verify all links to sequence records, if present, and make sure that each number retrieves the full record of the data. If a new accession number is not linked or a link is broken, provide Spectrum production staff with the correct URL for the record. If the accession numbers for new data are not publicly accessible before the expected online posting of the article, publication may be delayed; please contact production staff (Spectrum@asmusa.org) immediately with the expected release date.

Publication Fees: For information on publication fees and which article types are subject to charges, visit our website. If your manuscript is accepted for publication and any fees apply, you will be contacted separately about payment during the production process; please follow the instructions in that e-mail. Arrangements for payment must be made before your article is published.

ASM Membership: Corresponding authors may join or renew ASM membership to obtain discounts on publication fees. Need to upgrade your membership level? Please contact Customer Service at Service@asmusa.org.

The ASM Journals program strives for constant improvement in our submission and publication process. Please tell us how we can improve your experience by taking this quick Author Survey.

Thank you for submitting your paper to Spectrum.

Sincerely,
Travis Bourret
Editor
Microbiology Spectrum

Reviewer #1 (Comments for the Author):

The authors confirmed the long-held assumption that mouse infection by Borrelia burgdorferi can be accurately determined by culture of mouse tissues or by serology. I do not have any concerns over the technical aspects of this study or the manuscript

Reviewer #2 (Comments for the Author):

see attached

In this manuscript, Freeman-Gallant et al. describe the concordance of endpoints used to determine Lyme disease vaccine efficacy in mice. These include both culture and serology. While the study applies to a very specific niche of the field, the data are provided in a comprehensive and valuable way. It should be emphasized that these results are observed only in mice and are not observed in other animal models, nor are they likely to be identical in humans. Overall, this is well-written and the comparisons made are legitimate. Some modifications will improve the paper, and these are outlined below.

Introduction: line 69 “needle-mediated” is an awkward term here. Challenge by needle-inoculation of cultured Bb is more appropriate, though this could be eliminated altogether given that a Lyme vaccine must protect from tick challenge if it is to have value.

Line 85 should read “mouse serology.”

Results:

Line 93 should read “naïve,” instead of native.

The reference for a shorter C6 peptide (17mer vs 26mer) should be #19 Gomes-Solecki

Please clarify in the first paragraph that serology is IgG only.

Line 161: it is probably not correct to call these “false positives,” but rather are discordant results. The false-positive potential needs to be established using a pool of pre-immune or uninfected mouse sera to determine whether there is any reactivity to the OspC and DbpB antigens. If this has been done, it should be included or cited.

In Figure 1, please match the gray squares in the figure and legend.\

Figures 2 and 3. The numbers shown on the ROC curves are unclear. Usually, the sensitivity, specificity, AUC, and 95% CI are elaborated on the Figure itself.

Last paragraph before conclusions: it should be noted that the C6 portion of vlsE is not exposed on the surface of Borrelia and likely reactivity results from shed antigen on Borrelia that are not intact, whereas OspC and DbpB are surface lipoproteins. In this seroreactivity scenario, opsonization could have led to clearance, but retained exposure to the B cell response to surface-exposed antigens prior to clearance.

Conclusions: line 204. It is not clear how the assertion that “..collection of just the skin/ear tissues..is the most sensitive readout.” Was derived? From the Figures presented, it seems that the culture of other organs is very important as well.

Regarding limitations, the duration of post-challenge monitoring should be included. It is possible that more culture-positive tissues could emerge with >2 weeks time.

Methods: for culturing, please describe any measure of consistency used with respect to “a sample tissue of each of these organs..” used for culture. Effort to use the same size is necessary. Also please describe if the incubator used 5% CO_2 only or was microaerophilic.

Were all of the samples used in this study tested in the same lab with the same conditions? If not, this needs to be described.

Please consistently italicize the name of bacteria

Spectrum01446-26_R1 Response to Reviewers (R2R)

In this manuscript, Freeman-Gallant et al. describe the concordance of endpoints used to determine Lyme disease vaccine efficacy in mice. These include both culture and serology. While the study applies to a very specific niche of the field, the data are provided in a comprehensive and valuable way. It should be emphasized that these results are observed only in mice and are not observed in other animal models, nor are they likely to be identical in humans. Overall, this is well-written and the comparisons made are legitimate. Some modifications will improve the paper, and these are outlined below.

Comments (only R2 requested revisions)

1. Line 69 “needle-mediated” is an awkward term here. Challenge by needle inoculation of cultured Bb is more appropriate, though this could be eliminated altogether given that a Lyme vaccine must protect from tick challenge if it is to have value. **Response:** Text amended, as recommended.

2. Line 85 should read “mouse serology.” **Response:** Corrected

3. Line 93 should read “naïve,” instead of native. **Response:** Corrected

4. The reference for a shorter C6 peptide (17mer vs 26mer) should be #19 Gomes-Solecki. **Response:** Corrected

5. Please clarify in the first paragraph that serology is IgG only. **Response:** Corrected

6. Line 161: it is probably not correct to call these “false positives,” but rather are discordant results. The false-positive potential needs to be established using a pool of pre-immune or uninfected mouse sera to determine whether there is any reactivity to the OspC and DbpB antigens. If this has been done, it should be included or cited. **Response:** The Reviewer is spot on in the critique. To avoid confusion, we have removed reference to “true positive” and “true negative” outcomes and simply refer to them as culture positive/negative.

7. In Figure 1, please match the gray squares in the figure and legend. **Response:** Corrected as requested

8. Figures 2 and 3. The numbers shown on the ROC curves are unclear. Usually, the sensitivity, specificity, AUC, and 95% CI are elaborated on the Figure itself. **Response:** Corrected as requested

9. Last paragraph before conclusions: it should be noted that the C6 portion of vlsE is not exposed on the surface of Borrelia and likely reactivity results from shed

antigen on Borrelia that are not intact, whereas OspC and DbpB are surface lipoproteins. In this seroreactivity scenario, opsonization could have led to clearance, but retained exposure to the B cell response to surface-exposed antigens prior to clearance. Response: We thank the reviewer for pointing out this point. The text has been modified in several sections to underscore this point. For example, when first introducing the C6 peptide, we have inserted the following:

C6 is an appealing serological reagent because it is a derived from a highly conserved region within so-called invariable region 6 (IR6) of VlsE{Embers, 2007 #8354; Gomes-Solecki, 2007 #7808; Li, 2022 #8352}. However, it should be noted that C6 corresponds to an internal (buried) epitope that is presumably only accessible to B cell receptor recognition when VlsE is partially unfolded or degraded after being shed from the bacterial surface possibly at points after immune responses have been elicited against DbpB and OspC {Embers, 2007 #8354}.

10. Conclusions: line 204. It is not clear how the assertion that "..collection of just the skin/ear tissues..is the most sensitive readout." Was derived? From the Figures presented, it seems that the culture of other organs is very important as well. Response: That assertion is based on the results presented in Figure 1B, in which ear/skin cultures were positive in all but one infected animal (whose heart tissue was positive but not ES). We have modified the text to point this out.

11. Regarding limitations, the duration of post-challenge monitoring should be included. It is possible that more culture-positive tissues could emerge with >2 weeks time. Response: We note in the text that tissues were collected from mice three weeks after tick challenge and the cultures were monitored for a total of four weeks.

12. Methods: for culturing, please describe any measure of consistency used with respect to "a sample tissue of each of these organs.." used for culture. Effort to use the same size is necessary. Response: We have updated the text: Tissue samples for *B. burgdorferi* cultures were obtained from skin (2 mm punch biopsies), the entire bladder, ankles (bone segments ~ 3 mm proximal and distal to the joint after skin removal), and the cardiac apex (3 mm segment).

13. Also please describe if the incubator used 5% CO_2 only or was microaerophilic. Response: All cultures were performed in an incubator with 5% CO_2. We have noted this in the text.

14. Were all of the samples used in this study tested in the same lab with the same conditions? If not, this needs to be described. Response: Yes, we have now included the following in the text: Tick challenge studies were conducted in the same research laboratory under the same conditions.

15. Please consistently italicize the name of bacteria. **Response:** Corrected

Re: Spectrum01446-26R1 (**Serological correlates of disseminated *Borrelia burgdorferi* infection in mouse models of tick challenge**)

Dear Dr. Nicholas J. Mantis:

Your manuscript has been accepted, and I am forwarding it to the ASM production staff for publication. Your paper will first be checked to make sure all elements meet the technical requirements. ASM staff will contact you if anything needs to be revised before copyediting and production can begin. Otherwise, you will be notified when your proofs are ready to be viewed.

Data Availability: ASM policy requires that data be available to the public upon online posting of the article, so please verify all links to sequence records, if present, and make sure that each number retrieves the full record of the data. If a new accession number is not linked or a link is broken, provide production staff with the correct URL for the record. If the accession numbers for new data are not publicly accessible before the expected online posting of the article, publication may be delayed; please contact ASM production staff immediately with the expected release date.

Publication Fees: For information on publication fees and which article types have charges, please visit our website. We have partnered with Copyright Clearance Center (CCC) to collect author charges. If fees apply to your paper, you will receive a message from no-reply@copyright.com with further instructions. For questions related to paying charges through RightsLink, please contact CCC at ASM_Support@copyright.com or toll free at +1-877-622-5543. CCC makes every attempt to respond to all emails within 24 hours.

ASM Membership: Corresponding authors may join or renew ASM membership to obtain discounts on publication fees. Need to upgrade your membership level? Please contact Customer Service at Service@asmusa.org.

Embargo Policy: A press release may be issued as soon as the manuscript is posted on the Spectrum Latest Articles webpage. The corresponding author will receive an email with the subject line "ASM Journals Author Services Notification" when the article is available online.

Cover Image Submissions: If you would like to submit a potential Cover Image, please email a file and a short legend to spectrum@asmusa.org. Please note that we can only consider images that (i) the authors created or own and (ii) have not been previously published. By submitting, you agree that the image can be used under the same terms as the published article. Image File requirements: TIF/EPS, 7.5 inches wide by 8.25 inches tall (at least 2,250 pixels wide by 2,475 pixels tall), minimum 300 dpi resolution (600 dpi preferred), RGB, and no figure elements, e.g., arrows or panel labels. The legend should be a short description of the image, 1-2 sentences recommended. Please use the ASM Journals Cover Image Sizing Tool (https://journals.asm.org/pb-assets/html/asm-cover-sizing-tool.html) to ensure that your proposed cover image meets our size requirements.

The ASM Journals program strives for constant improvement in our submission and publication process. Please tell us how we can improve your experience by taking this quick Author Survey.

Thank you for submitting your paper to Spectrum.

Sincerely,
Travis Bourret
Editor
Microbiology Spectrum
